# Harnessing Total Scattering Techniques to Examine Local and Average Structure in Li/Mn Rich Cathodes

**DOI:** 10.1063/4.0001093

**Published:** 2025-10-27

**Authors:** Megan Murphy, Jue Liu, Boyu Shi, Gihan Kwon, Subhadip Mallick, Eungje Lee, Jason Croy, Michael Thackeray, Mahalingam Balasubramanian

**Affiliations:** 1 Oak Ridge National Laboratory, Oak Ridge, TN, USA; 2 Argonne National Laboratory, Lemont, IL, USA; 3 Brookhaven National Laboratory, Upton, NY, USA

## Abstract

A deep understanding of the structure of battery materials is crucial for achieving desired performance characteristics. Here, we outline the use of total scattering techniques as a powerful tool to investigate Earth-abundant cathode active materials (EaCAM). Just as X-rays and neutrons provide complementary insight, by examining both the local and average structure, total scattering gives rise to a comprehensive view of a material's atomic arrangement. These structural features are essential for understanding how the arrangement of atoms affects conductivity, cycling stability, and overall capacity. This talk will explore the structural changes that occur with varying ratios of Mn and Ni in lithium excess (LxS) Li2MnxNi2-xO4, focusing on the differences observed in the local and average structures as revealed through X-ray and neutron scattering techniques. Large-box modeling using RMCProfile will further elucidate these changes through multi-data fitting of the local atomic structure. By delving into the intricate interplay between local atomic environments and macroscopic properties, total scattering continues to be used as a powerful tool for optimizing next-generation energy storage systems.

